# Competing endogenous RNA expression profiling in pre-eclampsia identifies hsa_circ_0036877 as a potential novel blood biomarker for early pre-eclampsia

**DOI:** 10.1186/s13148-018-0482-3

**Published:** 2018-04-10

**Authors:** Xiaopeng Hu, Junping Ao, Xinyue Li, Huijuan Zhang, Ji Wu, Weiwei Cheng

**Affiliations:** 1 International Peace Maternity and Child Health Hospital, Shanghai Jiao Tong University School of Medicine, No. 910. Hengshan Road, Xuhui District, Shanghai, 200030 China; 2 Bio-X Institutes, Shanghai Jiao Tong University, No. 800. Dongchuan Road, Minhang District, Shanghai, 200240 China; 3 State Key Laboratory of Oncogenes and Related Genes, Shanghai Cancer Institute, Renji Hospital, Shanghai Jiao Tong University School of Medicine, Shanghai, 200032 China; 4 Key Laboratory of Fertility Preservation and Maintenance of Ministry of Education, Ningxia Medical University, Yinchuan, 750004 China

**Keywords:** Pre-eclampsia, ceRNA profiling, circRNA, Biomarker

## Abstract

**Background:**

The etiology and pathogenesis of pre-eclampsia (PE) is unclear, and there is no ideal early clinical biomarker for prediction of PE. The competing endogenous RNA (ceRNA) hypothesis is a new approach to uncover the molecular pathology of PE. The first aim of this study was to perform messenger RNA, long non-coding RNA, and circular RNA (circRNA) expression profiling of human normal and severe pre-eclampsia (SPE) placentas. circRNA, which has a stable structure, is a more suitable biomarker than other types of RNA. Therefore, the second aim of our study was to select some differentially expressed circRNAs in PE placentas as early clinical biomarkers of PE in blood circulation.

**Results:**

Using microarray analysis, we investigated differentially expressed ceRNAs in human normal and SPE placentas. Bioinformatics, such as gene ontology, KEGG pathway, and ceRNA network analyses, were performed to evaluate the microarray data and gain further insights into the biological processes. RNAs (*Chd5*, *Furin*, lnc-ELAVL4-9:1, lnc-RAP1GAP2-5:2, hsa_circ_0036877, hsa_circ_0036878, hsa_circ_0055724, hsa_circ_0049730, and hsa_circ_0036474) were validated by quantitative real-time PCR (qRT-PCR). RNA immunoprecipitation (RIP) of AGO2 in htra-8 cells and qRT-PCR analysis of hsa_circ_0036877 expression in maternal whole peripheral blood samples of participants were then conducted to confirm that hsa_circ_0036877 is a ceRNA and potential novel blood biomarker for early PE, respectively.

**Conclusion:**

Our study is the first systematic profiling of ceRNAs in placentas of PE patients and revealed the global ceRNA network integration in PE. Moreover, hsa_circ_0036877 can function as a ceRNA and serve as a potential novel blood biomarker for early PE.

**Electronic supplementary material:**

The online version of this article (10.1186/s13148-018-0482-3) contains supplementary material, which is available to authorized users.

## Background

At 20 weeks of gestation, pregnant women with high blood pressure and proteinuria are diagnosed with pre-eclampsia (PE) [1]. PE is one of the five kinds of gestational hypertension diseases, which can affect many organs and systems [2]. It occurs in about 2–8% of all pregnancies [3]. The etiology and pathogenesis of PE, which are unclear, may involve many factors such as trophoblast cell invasion [4], abnormal regulation of immune functions [5], endothelial cell injury [6], nutritional factors [7], and genetic factors [8, 9]. There is no single factor that can explain all causes and mechanisms of the disease. The central role of the placenta in the pathogenesis of PE is undisputed [10]. After termination of pregnancy, PE symptoms and complications are often relieved.

Many studies suggest that non-coding RNA participates in the pathogenesis of PE [11, 12]. Long non-coding RNA (lncRNA) and circular RNA (circRNA) can share microRNA (miRNA) response elements of linear messenger RNA (mRNA), which competitively bind to miRNAs, thus affecting the regulatory function of miRNAs and gene expression [13, 14]. The competing endogenous RNA (ceRNA) hypothesis suggests an interaction network between miRNAs, lncRNAs, circRNAs, and mRNAs. circRNAs can function as a ceRNA to regulate gene expression. Our study focused on ceRNAs, which may facilitate uncovering the etiology and pathogenesis of PE.

In the early stage of pregnancy before the onset of PE, prediction, effective intervention, prevention, and control are of great significance for the disease [15]. At present, there is no ideal early clinical biomarker for prediction of PE [16]. Although placental growth factor (PLGF), soluble fms-like tyrosine kinase-1 (sFlt-1), and placental protein 13 (PP13) have shown potential value, these molecules need further validation [17]. Non-coding RNA studies can reveal markers to predict PE. circRNA, a kind of non-coding RNA, is structured as a closed ring and does not have a free 5′ or 3′ end compared with traditional linear RNA [18]. This structure helps circRNA to avoid the action of RNA exonuclease, which allows it to maintain stable expression and makes it a more suitable biomarker than other RNA types [19, 20]. The aim of our study was to identify a differentially expressed circRNA in PE placentas as an early clinical biomarker of PE in blood circulation.

## Methods

### Study design and population

This is a prospective cohort nest study. In the study, we recruited a total of 1000 “healthy” pregnant women (age from 25 to 35) followed until delivery and maternal whole peripheral blood samples were collected at 24 weeks of gestation. In all, 40 patients with PE were included in the study and 116 normal healthy women were matched based on maternal age and gestation age. This study was divided into two phases: ceRNA profiling research (discovery research) and biomarker study (prospective development research). In the first phase, we included 6 patients with severe pre-eclampsia (SPE) and 6 matched healthy pregnant women. The second phase was the prospective development study on hsa_circ_0036877 of maternal whole peripheral blood to predict PE at 24 weeks of gestation. The rest participants (34 patients with PE and 110 matched normal healthy women) were included in the phase 2 study. The study design is shown in Additional file 1: Figure S1.

### Samples and setting

The study was approved by the Research Ethic Committees at the International Peace Maternity and Child Health Hospital of China Welfare Institute, SJTUM, China. A total of 110 placentas and 1000 maternal whole peripheral blood samples in the study were collected from participants (Chinese women) between December 2015 and March 2017 at the International Peace Maternity and Child Health Hospital of China Welfare Institute (Shanghai, China). Placental samples were taken from a representative block of the central portion of tissue below one third of the placenta, near maternal side. All samples were taken from the same region. This region was enriched for cytotrophoblast, syncytiotrophoblast, and villous interstitium. Samples were stored at − 80 °C until use. The demographic characteristics of participants are shown in Additional file 1: Tables S1 and S2. Forty PE patients included in the study without a history of hypertension, diabetes, or kidney disease were evaluated by an experienced obstetrician. According to the American College of Obstetricians and Gynecologists (ACOG), PE is defined as systolic blood pressure of ≥ 140 mmHg and diastolic blood pressure of ≥ 90 mmHg with proteinuria of ≥ 300 mg/day (or a protein/creatinine ratio of ≥ 0.3 mg/dl or proteinuria of ≥ 1+) or without proteinuria but with severe clinical features after 20 weeks of gestation in a woman with previously normal blood pressure [21]. Diagnosis criteria of SPE were as follows: blood pressure of ≥ 160/110 mmHg with proteinuria of ≥ 300–5000 mg/day.

### RNA extraction and purification

Total RNA was extracted from placentas and whole blood using RNAiso Plus (Cat# 9109; TaKaRa) following the manufacturer’s instructions and checked for a RNA integrity number (RIN) to inspect RNA integrity by an Agilent Bioanalyzer 2100 (Agilent Technologies, Santa Clara, CA, USA). RIN ≥ 7.0 and 28 s/18 s ≥ 1.9. Qualified total RNA was further purified by an RNeasy mini kit (Cat# 74106; QIAGEN, GmBH, Germany) and RNase-Free DNase Set (Cat# 79254; QIAGEN).

### RNA amplification and labeling

RNA was amplified and labeled by a Low Input Quick Amp WT Labeling Kit (Cat# 5190-2943; Agilent Technologies) following the manufacturer’s instructions. Labeled cRNA was purified by the RNeasy mini kit.

### Quality control of the microarray

Coefficient of variation (CV) method was applied to the microarray quality control. CV, which was defined as the ratio of the standard deviation to the average, was expressed in percentage. The calculation formula is CV = Standard Deviation (SD) / Mean × 100%. The comparative analysis between the two sets of CV value data determined whether the system is stable or not. In Agilent expression profile chip experiments, the CV value of repeated probe point (repeat 10 times) signals calculated the stability of the chip and technology. According to the different chips, the probe point signals were repeated from 10 to 100 times.

Calculation method of chip detection rate: The ratio of the total number of points (flags which does not contain A) to the total number of probes is the detection rate of the chip.

### Microarray analysis

Each slide was hybridized with 1.65 μg Cy3-labeled cRNA using a Gene Expression Hybridization Kit (Cat# 5188-5242; Agilent Technologies) in a Hybridization Oven (Cat# G2545A; Agilent Technologies) according to the manufacturer’s instructions. After 17 h of hybridization, the slides were washed in staining dishes (Cat# 121; Thermo Shandon, Waltham, MA, USA) with a Gene Expression Wash Buffer Kit (Cat# 5188-5327; Agilent Technologies) following the manufacturer’s instructions.

Slides were scanned by an Agilent Microarray Scanner (Cat# G2565CA; Agilent Technologies) with default settings (dye channel: green; scan resolution = 3 μm; PMT: 100%, 20 bits). Data were extracted with Feature Extraction software 10.7 (Agilent Technologies). Raw data were normalized by the quantile algorithm of limma package in R. The Raw data is available and deposited in public database (GEO). The GEO accession number is GSE102897.

### Bioinformatics analysis

Hierarchical clustering was performed in MeV_4_9_0 to identify and visualize patterns within the microarray dataset. Gene ontology (GO) and Kyoto Encyclopedia of Genes and Genomes (KEGG) pathway analyses were performed using GOseq and KOBAS, respectively. The *p* value was calculated using a right-side hypergeometric test. The Benjamini-Hochberg adjustment was used to correct for multiple tests. An adjusted *p* value of < 0.05 indicated a statistically significant deviation from the expected distribution. All differentially expressed RNAs were evaluated by GO and KEGG pathway analyses. The network visualization and analysis tool Cytoscape_V2_8_3 was used to identify putative target genes for the predicted miRNA sponge.

### Quantitative real-time (qRT)-PCR

cDNA was synthesized from 1 μg of total RNA using HiScript reverse transcriptase (HiScript II 1st Strand cDNA synthesis kit; Vazyme, China) in a 20-μl volume containing reverse transcription primer, 25 μM oligo(dT), and 10 μM random primers. qRT-PCR was carried out with FastStart Universal SYBR Green Master (Rox) (Roche Diagnostics, Indianapolis, IN, USA) in a 10-μl reaction volume on a 7500 Real-Time PCR System using the following conditions: 95°C for 30 s, followed by 40 cycles of 95°C for 5 s and 60°C for 34 s, and then 95°C for 15 s, 60°C for 60 s, and 95°C for 15 s. 0.1 μg RNA was used for all qRT-PCR experiments. For all qRT-PCR experiments, *Gapdh* was the control gene. Data analysis was performed by the Δ cycle threshold (ΔCt) method. ΔCt = CT_RNA_ − CT_*Gapdh*_, which represents the amount of RNAs normalized relative to the amount of *Gapdh*. qRT-PCR primers from Generay Biotech Co., Ltd. (Shanghai, China) are listed in Additional file 1: Table S4.

### RNA immunoprecipitation (RIP)

RIP experiments were performed using a Magna RIP RNA-Binding Protein Immunoprecipitation Kit (Millipore, Bedford, MA, USA). The AGO2-RIP assay was conducted in htra-8 cells. Approximately 1 × 10^7^ cells were pelleted and re-suspended with an equal volume of RIP lysis buffer (about 100 μl) containing protease and RNase inhibitors. Cell lysates (100 μl) were incubated with 5 μg control rabbit IgG- or anti-AGO2 antibody (Abcam)-coated beads with rotation at 4 °C overnight. After treating with proteinase K, the immunoprecipitated RNAs were extracted by an RNeasy MinElute Cleanup Kit (QIAGEN) and reverse transcribed using Prime-Script RT Master Mix (TaKaRa). The level of hsa_circ_0036877 was detected by qRT-PCR.

### RNA fluorescence in situ hybridization (RNA FISH)

Oligonucleotide-modified probe sequence for hsa_circ_0036877 (GGGACTATGCAAACCAGGGGTGCGCATGCTGGAT) was applied for FISH. First, the probe of hsa_circ_0036877 was marked with BIOTIN-UTP (MEGAshortscript™ Kit, AM1354) for RNA labeling. The htra-8 cells were cultured in 24-well, and the placenta tissue was frozen in OCT and sectioned 5 μm, which were washed with PBS and fixed in 4% paraformaldehyde. After hybridization, slides were washed three times with PBS, were incubated with HRP-conjugated streptavidin to the cells or tissue, and were incubated for 60 min at room temperature (Thermo Fisher scientific, MAN0015834). After being washed for three times for 10 min at room temperature, the slides were incubated with Alexa Fluor™488 tyramide reagent (Thermo Fisher Scientific, MAN0015834) for 10 min and were sealed with tablets containing DAPI. The images were acquired using a fluorescence microscopy (Leica).

### Hematoxylin and eosin (HE) assay

Placenta samples were fixed in 4% paraformaldehyde overnight. Then, the tissues were dehydrated in a graded ethanol series, cleared in a xylene solution, and embedded in paraffin. The paraffin-embedded tissues were sectioned at 5-μm thicknesses. The sections were dewaxed, hydrated, and then stained with hematoxylin and eosin. For the assessment of apoptosis in placenta, 10 high-power fields (× 100 magnifications) were selected randomly in each specimen (from the same individuals in the PE (*n* = 40) and non-PE groups (*n* = 33)), and apoptosis of trophoblastic cells was examined. The number of apoptosis cells/field was counted to represent the apoptotic index.

### Statistical analyses

Statistical analyses were performed using GraphPad Prism 5.0 and Statistical Program for Social Science version 22 (SPSS 22.0). Statistically significant differences were calculated by the Student *t* test. A receiver operating characteristic (ROC) curve was established for hsa_circ_0036877 using SPSS 22.0 to determine whether it was an ideal blood biomarker for early PE.

## Results

### Differentially expressed mRNAs, lncRNAs, and circRNAs, and cluster analysis

**Fig. 1:**
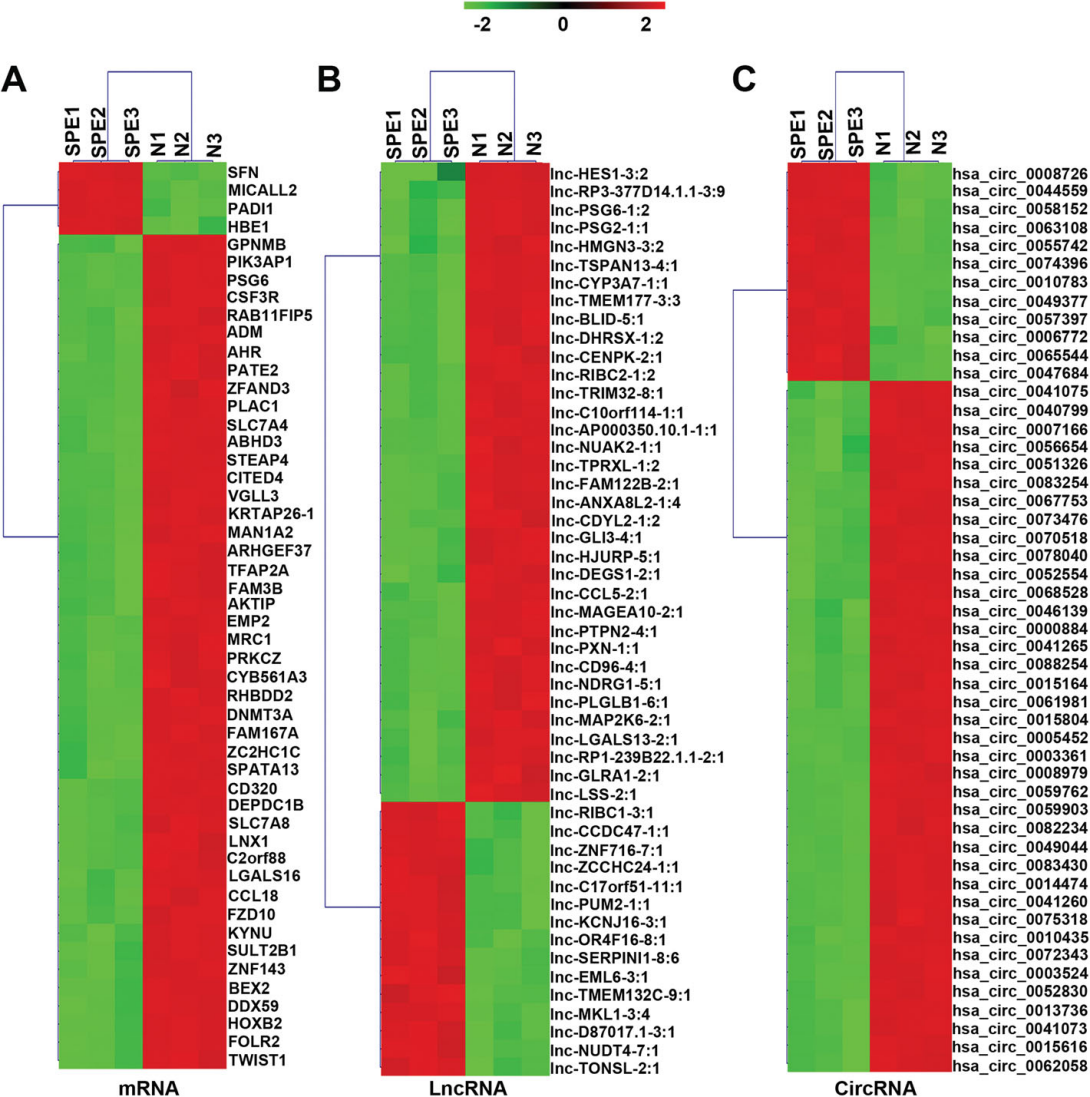
Hierarchical clustering of mRNAs (**a**), lncRNAs (**b**), and circRNAs (**c**) in placenta samples between SPE and normal controls. Placenta samples were clustered based on the expression profiles of 50 twofold differentially expressed mRNAs, 50 twofold differentially expressed lncRNAs, and 50 twofold differentially expressed circRNAs. The color bar, which increases from green to red comparing SPE samples with matched normal controls, indicates mRNA, lncRNA, and circRNA expression levels

Because of the central role of the placenta in PE pathogenesis, we first performed microarray analysis of six placenta samples from SPE patients and six placenta samples from matched normal controls (*N*). After applying a stringent filtering approach that compared SPE and *N* groups (adjusted *p* value, < 0.05; fold change, > 2 or < 0.5), we identified 731 upregulated and 1617 downregulated mRNAs, 5127 upregulated and 3813 downregulated lncRNAs, and 4569 upregulated and 3984 downregulated circRNAs. The top 10 upregulated and downregulated circRNAs are shown in Additional file 1: Table S3. Moreover, based on these differentially expressed mRNAs, lncRNAs, and circRNAs, a tree with a clear distinction between SPE and *N* groups was generated by MeV_4_9_0 cluster analysis (Fig. 1). The variability between controls in microarray vs variability between PE samples was shown in BOX plot (Additional file 1: Figure S2). These results suggested that ceRNA expression in the SPE group can be robustly separated from that in the *N* group.

### GO enrichment of differentially expressed mRNAs, lncRNAs, and circRNAs

**Fig. 2:**
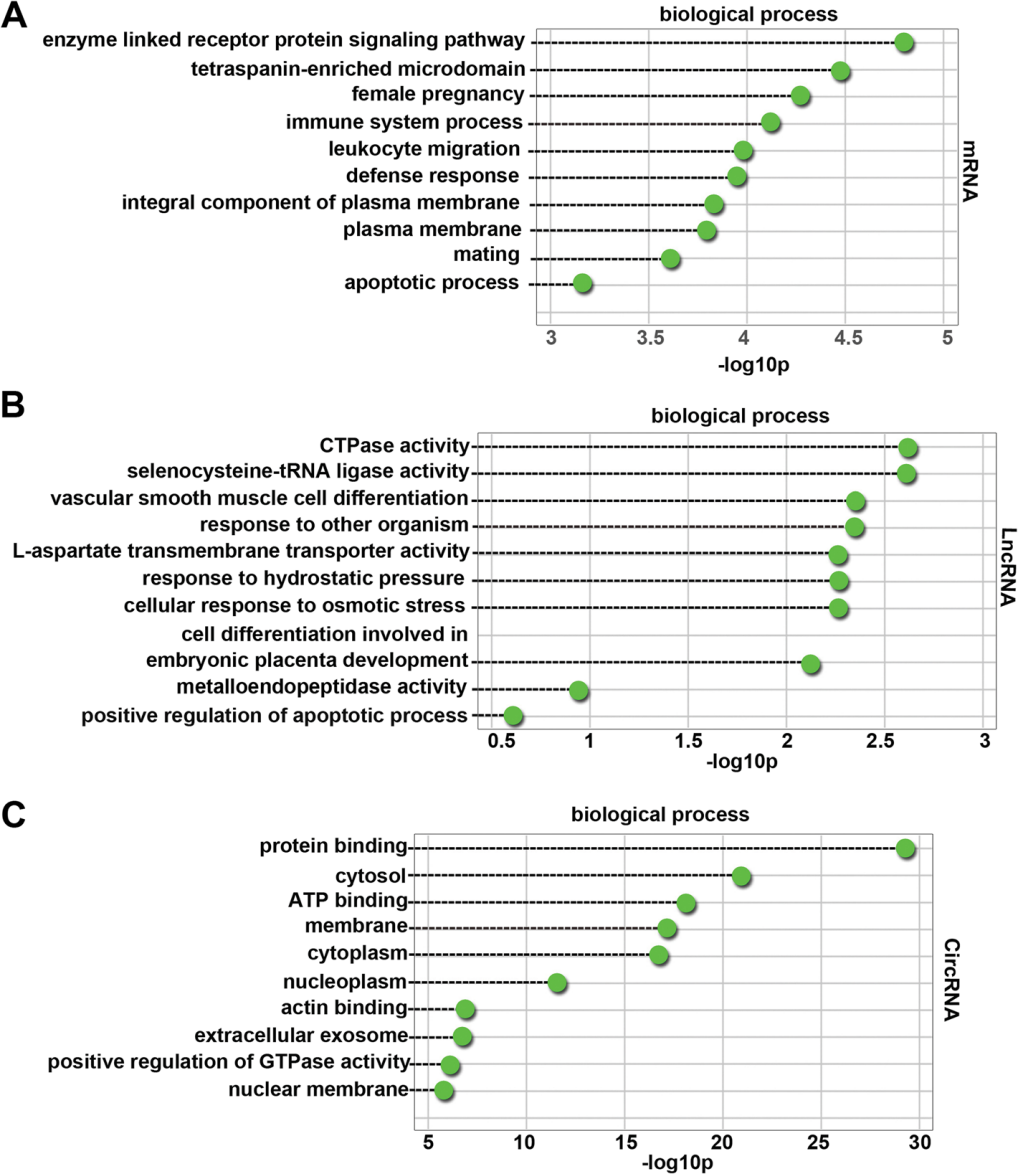
Top 10 categories of GO biological processes associated with differentially expressed mRNAs (**a**), lncRNAs (**b**), and circRNAs (**c**). Dashed lines represent the *p* values of the top 10 GO biological processes. The *p* values were calculated by hypergeometric tests and corrected by the Benjamini-Hochberg adjustment. *p* values are expressed as the negative logarithm (base 10)

We identified the functional categories of these differentially expressed mRNAs, lncRNAs, and circRNAs by performing GO analysis to gain further insights into the biological processes that were potentially mediated by these differentially expressed molecules in PE. For differentially expressed mRNAs, 217 GO terms in the category of biological processes were significantly enriched at a false discovery rate (FDR) threshold of < 0.05. As shown in Fig. 2a, the top 10 significantly enriched biological processes were enzyme-linked receptor protein signaling pathway, tetraspanin-enriched microdomain, female pregnancy, immune system process, leukocyte migration, defense response, integral component of plasma membrane, plasma membrane, mating, and apoptotic process. Many biological processes were associated with the pathogenesis of PE, such as immune system and apoptotic processes.

There were 72 significantly enriched GO terms (FDR threshold of < 0.05) for differentially expressed lncRNAs. The top 10 significantly enriched biological processes are shown in Fig. 2b. They were CTPase activity, selenocysteine-tRNA ligase activity, vascular smooth muscle cell differentiation, response to other organism, l-aspartate transmembrane transporter activity, response to hydrostatic pressure, cellular response to osmotic stress, cell differentiation involved in embryonic placenta development, metalloendopeptidase activity, and positive regulation of apoptotic processes. Many biological processes were related to the mechanisms of PE, such as cell differentiation involved in embryonic placenta development and metalloendopeptidase activity. Four hundred and forty-four GO terms were significantly enriched (FDR threshold of < 0.05) for host genes of differentially expressed circRNAs. As shown in Fig. 2c, the top 10 significantly enriched biological processes were protein binding, cytosol, ATP binding, membrane, cytoplasm, nucleoplasm, actin binding, extracellular exosome, positive regulation of GTPase activity, and nuclear membrane. The most abundant categories were those associated with pathological processes that may induce damage in the membrane, cytoplasm, nucleoplasm, and nuclear membrane of placental cells.

### KEGG enrichment of differentially expressed mRNAs, lncRNAs, and circRNAs

**Fig. 3:**
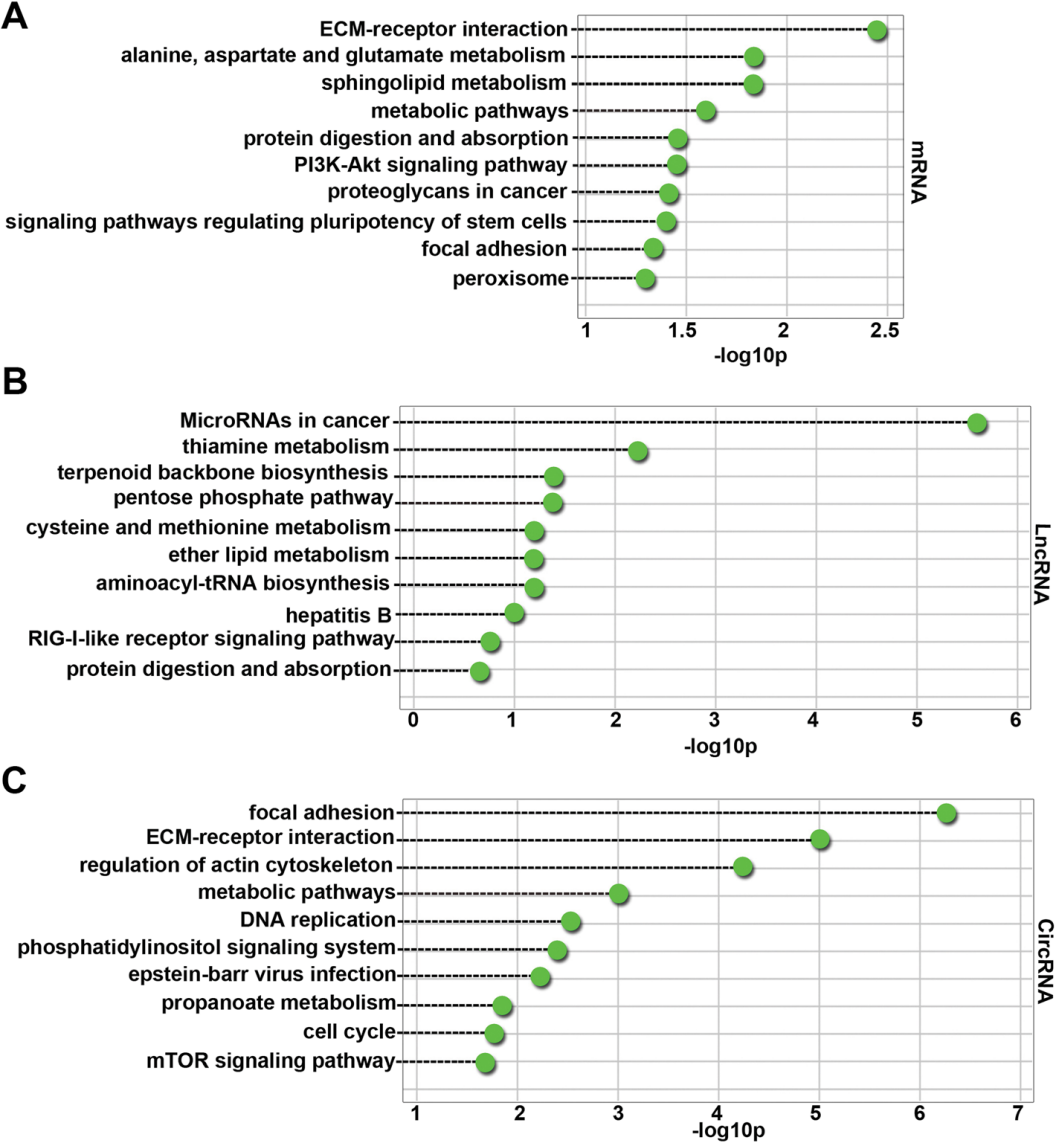
Top 10 pathways significantly enriched in differentially expressed mRNAs (**a**), lncRNAs (**b**), and circRNAs (**c**). Dashed lines represent the *p* values of the significantly enriched top 10 GO pathways. The *p* values were calculated by hypergeometric tests and corrected by the Benjamini-Hochberg adjustment. *p* values are expressed as the negative logarithm (base 10)

Ninety-four KEGG pathways (FDR *p* value < 0.05) were associated with differentially expressed mRNAs. As shown in Fig. 3a, the top 10 enriched pathways were ECM-receptor interaction, alanine, aspartate and glutamate metabolism, sphingolipid metabolism, metabolic pathways, protein digestion and absorption, PI3K-Akt signaling pathway, proteoglycans in cancer, signaling pathways regulating pluripotency of stem cells, focal adhesion, and peroxisome. In particular, a study has shown that the phosphatidylinositide 3-kinase (PI3K)-Akt signaling pathway is associated with PE [22]. Therefore, the roles of the PI3K-Akt signaling pathway in human PE placentas were investigated further.

There were 14 KEGG pathways (FDR *p* value < 0.05) associated with differentially expressed lncRNAs. As shown in Fig. 3b, the top 10 enriched pathways were microRNAs in cancer, thiamine metabolism, terpenoid backbone biosynthesis, pentose phosphate pathway, cysteine and methionine metabolism, ether lipid metabolism, aminoacyl-tRNA biosynthesis, hepatitis B, RIG-I-like receptor signaling pathway, and protein digestion and absorption. These results indicated that differentially expressed lncRNAs in PE control gene expression on post-transcription level. One hundred and fifty-three KEGG pathways (FDR *p* value < 0.05) were associated with host genes of differentially expressed circRNAs. As shown in Fig. 3c, the top 10 enriched pathways were focal adhesion, ECM-receptor interaction, regulation of actin cytoskeleton, metabolic pathways, DNA replication, phosphatidylinositol signaling system, Epstein-Barr virus infection, propanoate metabolism, cell cycle, and the mammalian target of rapamycin (mTOR) signaling pathway. These results indicated that host genes of differentially expressed circRNAs in PE were involved in the mTOR signaling pathway that is related to angiogenic factor expression in the placenta of rats [23].

### Validation of mRNA, lncRNA, and circRNA expression

**Fig. 4:**
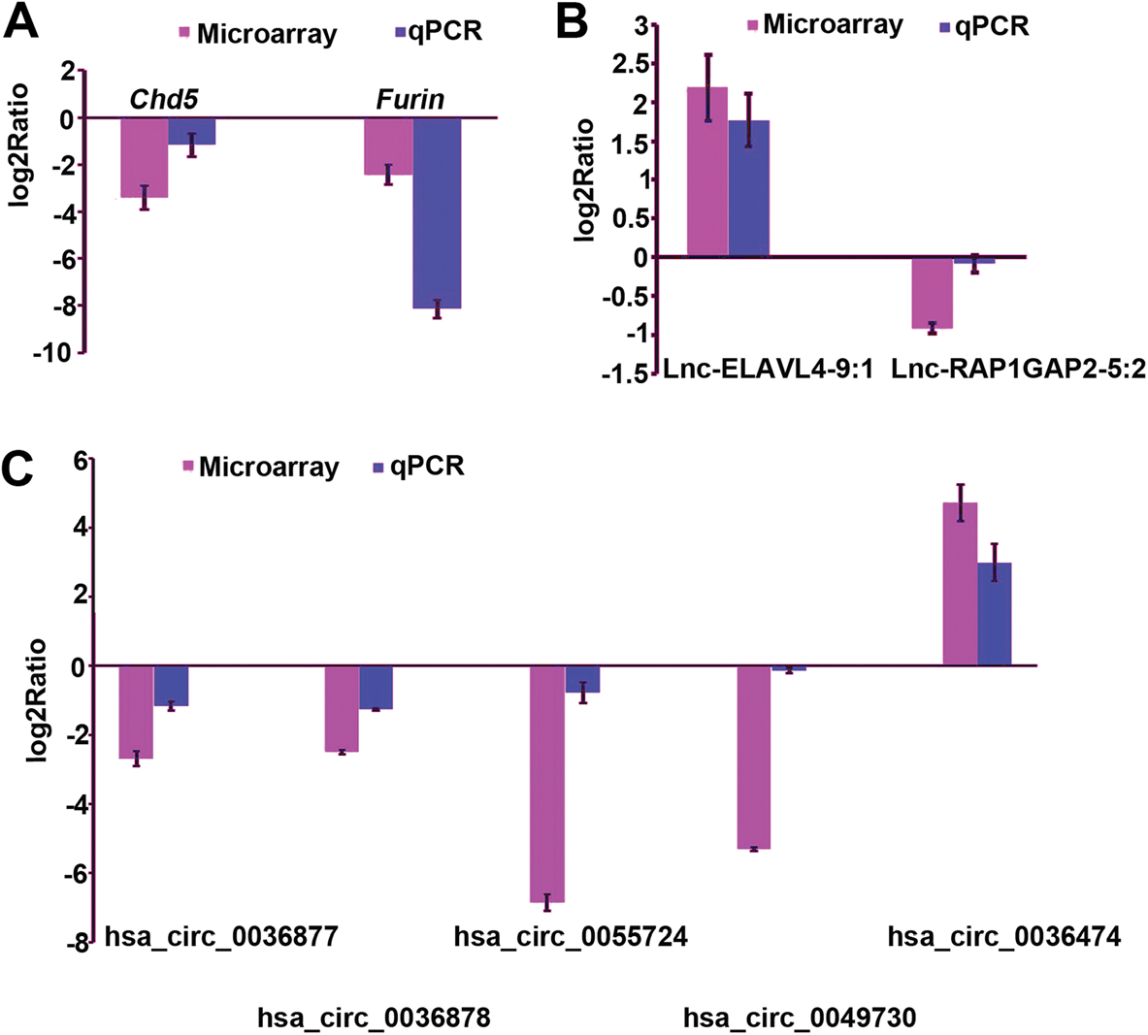
mRNA (**a**), lncRNA (**b**), and circRNA (**c**) expression validated by qRT-PCR. Genes determined to be differentially expressed in all SPE patients by microarray analysis were validated by qRT-PCR. The height of the columns in the chart represents the log-transformed average fold change in expression across the two groups of patients for each of the validated genes. Bars represent standard errors

Some mRNAs (*Chd5* and *Furin*), lncRNAs (lnc-ELAVL4-9:1 and lnc-RAP1GAP2-5:2), and circRNAs (hsa_circ_0036877, hsa_circ_0036878, hsa_circ_0055724, hsa_circ_0049730, and hsa_circ_0036474) identified as differentially expressed by the microarray analysis were selected for validation (Fig. 4). qRT-PCR was performed to detect the expression of these mRNAs, lncRNAs, and circRNAs in PE (*n* = 10) and *N* (*n* = 10) placenta samples. As shown in Fig. 4, the qRT-PCR results were highly consistent with the microarray data. Melting curve analysis and agarose gel electrophoresis were used to check for the specificity of the qRT-PCR products.

### Global ceRNA network integration in PE

**Fig. 5:**
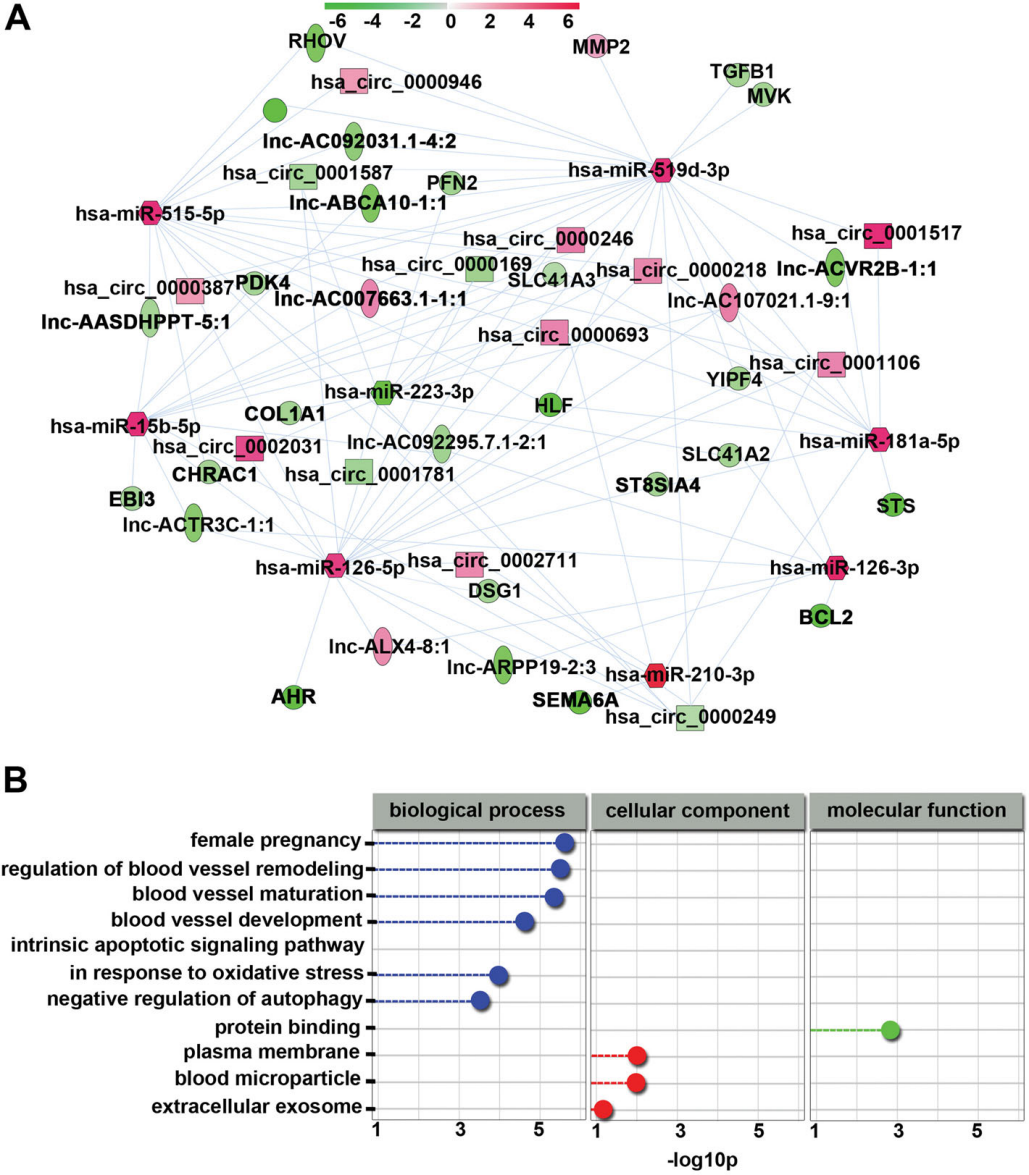
Global ceRNA network integration in PE. **a** A portion of the theoretical ceRNA network in PE according to the microarray analysis and report data was predicted. The color bar, which increases from green to red comparing SPE samples with matched normal controls, indicates mRNA, lncRNA, and circRNA expression levels. **b** GO biological processes of the above module were involved in the pathogenesis of PE. Blue nodes represent biological processes, red nodes represent cellular components, and green nodes represent molecular functions. *p* values were calculated by hypergeometric tests and corrected using the Benjamini-Hochberg adjustment. *p* values are expressed as negative logarithms (base 10)

Previous studies show that miRNAs (miR-210 [24], miR-181a [25], miR-126-3p and miR-126-5p [26], miR-223 [27], miR-515 [28], and miR-519d [29]) are involved in the pathogenesis of PE. Based on our microarray data and significant expression of miRNAs associated with PE in previous reports, a global ceRNA network was predicted using Cytoscape_V2_8_3. As shown in Fig. 5a, a portion of the global ceRNA network was observed and GO analysis was performed to gain further insights into the biological processes in this module. Remarkably, we observed that the target genes of this module were implicated in biological processes and molecular functions in the pathogenesis of PE, such as blood vessel maturation and angiogenesis (Fig. 5b).

### Hsa_circ_0036877 can function as a ceRNA

The proprotein convertase Furin, which is required for the development of the syncytiotrophoblast structure in the labyrinth layer and normal embryonic development [30, 31], is the host gene of hsa_circ_0036877. In view of this, we explored whether has_circ_0036877 can function as a ceRNA in htra-8 cells (the htra-8 cell derived from first trimester human placenta was cell line of trophoblasts).

Firstly, we investigated the expression of hsa_circ_0036877 in placenta of patients with PE and htra-8 cells by RNA FISH. As shown in Additional file 1: Figure S3A and B, hsa_circ_0036877 was high expressed in cytoplasm of syncytial trophoblasts (arrows show) and was higher expressed in normal placenta than PE. Next, to detect the ability of hsa_circ_0036877 to sponge miRNAs, endogenous hsa_circ_0036877 binding of Ago2 was determined by RNA-binding protein immunoprecipitation (RIP) in htra-8 cells. RIP assay showed that the amount of endogenous hsa_circ_0036877 pulled-down using AGO2 antibody was detected by qRT-PCR and significantly higher than control using IgG antibody (Additional file 1: Figure S3C). This experiment can identify that hsa_circ_0036877 might recruit AGO2 to sponge miRNAs, because the “minimal RNA-induced silencing complex (RISC)” appears to include AGO2 bound to a short guide RNA such as a miRNA or short interfering RNA (siRNA), which direct RISC to complementary mRNAs and silence gene expression [32].

Furthermore, as predicted by Cytoscape_V2_8_3, hsa_circ_0036877 may function as a ceRNA to bind to miR-519d-3p and miR-15b-5p, thus affecting gene expression (Additional file 1: Figure S3D). GO analysis in this module was performed to gain further insights into the biological processes (Additional file 1: Figure S3E). It is worth noting that biological processes and molecular functions in the pathogenesis of PE, such as regulation of blood vessel remodeling and female pregnancy, were enriched in this module. These results suggested that hsa_circ_0036877 may function as a ceRNA and sponge miRNAs, thus implicating it in the pathogenesis of PE.

### Hsa_circ_0036877 can serve as a potential novel blood biomarker for early PE

**Fig. 6:**
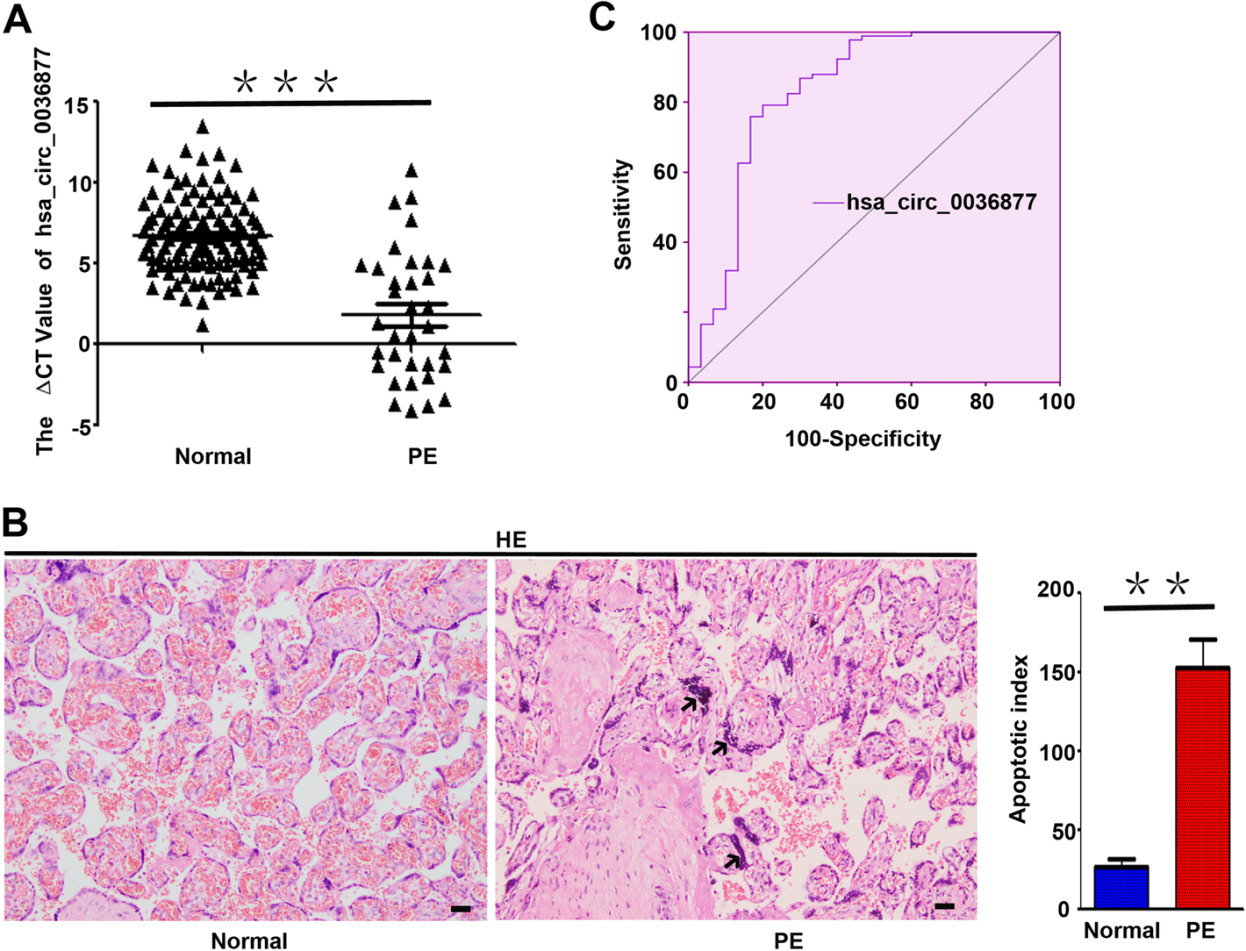
Hsa_circ_0036877 can serve as a potential novel blood biomarker for early PE. **a** Expression of hsa_circ_0036877 in blood samples from 110 matched normal participants and 34 patients with PE at 24 weeks of gestation was detected by qRT-PCR. The expression of hsa_circ_0036877 in blood samples of PE (ΔCt mean ± s.e.m., 1.778 ± 0.6888) diagnosed after gestation based on ACOG was significantly higher than in normal controls (ΔCt mean ± s.e.m., 6.651 ± 0.2192). Higher ΔCt value indicated lower expression. Bars indicate means ± s.e.m. from independent experiments. ***p < 0.0001. **b** Compared with matched normal controls, significantly increased apoptosis of syncytial trophoblasts was found in the PE placenta. Right panel showed that the apoptosis index in normal and PE placenta was statisticed. Scale bar, 50 μm. **p < 0.001 **c** ROC curves showed that hsa_circ_0036877 can serve as a potential blood biomarker for prediction of PE. AUC, 0.846 (95% CI 0.754–0.938); sensitivity and specificity, 85.3 and 72.7%, respectively. The cutoff value was 5.13 (ΔCt value) (the optimal cutoff point was determined at the maximum of Youden index (YI)). The PPV of an ΔCt value of 5.13 or lower for a diagnosis of preeclampsia was 49.1%. An ΔCt value above 5.13 had a high NPV of 94.1%

Hsa_circ_0036877 expression was lower in placenta samples of PE patients (Fig. 4c). To confirm that differentially expressed circRNAs in microarray data can serve as blood biomarkers for early PE, we selected hsa_circ_0036877 whose expression in maternal whole peripheral blood from 110 matched normal participants and 34 patients with PE at 24 weeks of gestation was detected by qRT-PCR. The qRT-PCR results showed that expression of hsa_circ_0036877 was significantly higher in blood samples of 34 patients with PE (ΔCt mean ± s.e.m., 1.779 ± 0.6888) than in those of 110 normal participants (ΔCt mean ± s.e.m., 6.651 ± 0.2192), which were diagnosed at the end of gestation based on ACOG (Fig. 6a). Higher ΔCt value indicated lower expression. Furthermore, Additional file 1: Figure S4 shows the correlation between the expression in placenta and maternal blood from the same individuals in the PE (*n* = 40) and non-PE groups (*n* = 33). These results indicated that hsa_circ_0036877 expression was lower in placenta samples of PE, but higher in whole peripheral blood samples of patients with PE than normal controls.

Leading hypotheses suggest that necrotic breaks occur in the syncytiotrophoblast as a direct consequence of an imbalance in the regulation of apoptosis, leading to increased release of trophoblast particles (TPs) into maternal circulation in PE [33]. Extracellular nucleic acids of both fetal and placental origin, packed into either trophoblast-derived apoptotic bodies or shedding syncytiotrophoblast microparticles, which are significantly increased in PE, may be detected in maternal circulation [34, 35]. In our study, hsa_circ_0036877 may be one of the extracellular nucleic acids originating from the placenta. As shown in Fig. 6b, the significantly increased apoptosis of syncytial trophoblasts (arrows) can explain the phenomenon that lower expression of hsa_circ_0036877 in PE placentas, but higher expression in blood circulation.

Furthermore, the area under the receiver operating characteristic (ROC) curve (AUC) of hsa_circ_0036877 was 0.846 [95% confidence interval (CI) 0.754–0.938]. The cutoff value (the optimal cutoff point was determined at the maximum of Youden index (YI)) was 5.13 (ΔCt value) (Fig. 6c). The sensitivity and specificity were 85.3 and 72.7%, respectively. The positive predictive value (PPV) of an ΔCt value of 5.13 or lower for a diagnosis of preeclampsia was 49.1%. An ΔCt value above 5.13 had a high negative predictive value (NPV) of 94.1%. These results suggested that hsa_circ_0036877 may have some predictive value for early PE.

## Discussion

### Main findings

In this study, we identified the ceRNA network by integrated analyses of mRNA, lncRNA, and circRNA expression profiles in human normal and SPE placentas. The global ceRNA network was predicted based on microarray data, and hsa_circ_0036877 functions as a ceRNA, thus affecting gene expression in trophoblast cells.

The second aim of our study was to identify differentially expressed circRNAs in PE placentas as early clinical biomarkers of PE in blood circulation. In prospective development study, qRT-PCR showed that the expression of hsa_circ_0036877 was significantly higher in blood samples from PE patients than normal participants. Furthermore, the ROC analyses of qRT-PCR results indicated that hsa_circ_0036877 was a potential blood biomarker for early PE.

### Strength and limitations

We investigated differentially expressed ceRNAs in PE placenta samples compared with normal placenta samples by Agilent microarray analysis and qRT-PCR. The results of the microarray analysis were highly consistent with those of qRT-PCR in our study. The qRT-PCR was used to confirm the ceRNA expression profiles obtained from the microarray data. Although qRT-PCR provides higher accuracy and sensitivity than microarray, qRT-PCR data only represent one point data and microarray data can represent overall present state of participants. So microarray seems to over represent data for ceRNA compared to qRT-PCR.

Recent studies have identified some differentially expressed circRNAs in blood circulation [36, 37] and PE placentas [38] using Agilent and KangChen circRNA microarrays, respectively. Because there are different gene probes between Agilent (approximately 90,000 probes) and KangChen (301 probes) circRNA microarrays (e.g., there are no expression probes for hsa-circRNA-100782, hsa-circRNA-102682, or hsa-circRNA-104820 in the Agilent circRNA microarray) [38], our findings did not conflict with their results.

In previous studies, mRNA [39] and lncRNA [40] expression profiling of PE placentas was performed. We first systematically elucidated the CeRNA expression profiles of the placentas of PE patients and characterized the genetic pathways. Systematic comparisons demonstrated common pathological changes in the placenta of patients with PE. Molecular processes and signaling pathways controlling cell fates (e.g., the cell cycle and mTOR signaling pathway) were found to be significantly disrupted in PE based on microarray analysis. These results provide insights into the pathoetiology of PE as well as reproductive fitness at the molecular level and suggest a series of targets for therapy.

Our study has some limitations. Firstly, due to time, region, and economic problems, we investigated Chinese population, but other population was not done in this paper. Secondly, an important limitation of the study is the relatively small sample size. We will set multi-centers and recruit much more participants to investigate the hsa_circ_0036877 of biomarker research to predict PE in next project study.

### Interpretation

Pre-eclampsia is the leading cause of pregnancy-associated morbidity and mortality, and the management of this complex syndrome needs to be improved [41–43]. Although the etiology and pathogenesis of PE are unclear, competing endogenous RNA (ceRNA) hypothesis is a new approach to uncover the molecular pathology of PE. Studies have revealed that non-coding RNA is involved in the pathogenesis of PE [11, 12], and further implicated that ceRNA may play role in its processes. CeRNA profiling and network analyses of SPE placentas facilitated identification of deregulated biological processes or pathways that might be regulated by ceRNAs that could serve as ideal therapeutic targets. Based on ceRNA microarray data and RIP assay, we predicted potential targets of hsa_circ_0036877, which suggested that hsa_circ_0036877 may be related to PE. Next, the ceRNA mechanism of hsa_circ_0036877 in PE need to be further investigated in vivo and in vitro.

The tissue specificity and stability of circRNA enhance its potential for use as a biomarker for the diagnosis of PE [44]. The area under the receiver operating characteristic (ROC) curve (AUC) of hsa_circ_0036877 was 0.846 [95% confidence interval (CI) 0.754–0.938]. An ΔCt value above 5.13 had a high NPV of 94.1%. Hsa_circ_0036877 may have some early predictive value for PE and need to be further validated.

## Conclusion

Our study is the first systematic profiling of ceRNAs in placentas of PE patients and revealed the global ceRNA network integration in PE. Moreover, hsa_circ_0036877 can function as a ceRNA and serve as a potential novel blood biomarker for early PE.

## Additional file

Additional file 1: The demographic characteristics of  the pregnant women which 12 placentas were from. The demographic characteristics of the pregnant women which blood samples were from. The top 10 differentially expressed circRNAs in placenta of PE using microarray analyses. Oligonucleotide primer sequences for qRT-PCR. Study disign. The variability between controls in microarray vs variability between PE samples was shown in BOX plot. Hsa_circ_0036877 can function as a ceRNA in the PE placenta. The correlation between the expression in placenta and maternal blood from the same individuals in the PE and non-PE grounps.  (PDF 967 kb)

## Abbreviations

ACOG: American College of Obstetricians and Gynecologists
AUC: Area under the receiver operating characteristic curve
BMI: Body mass index
CeRNA: Competing endogenous RNA
CI: Confidence interval
circRNA: Circular RNA
CV: Coefficient of variation
FDR: False discovery rate
GO: Gene ontology
HE: Hematoxylin and eosin
KEGG: Kyoto Encyclopedia of Genes and Genomes
lncRNA: Long non-coding RNA
miRNA: Micro-RNA
mRNA: Messenger RNA
NPV: Negative predictive value
PE: Pre-eclampsia
PLGF: Placental growth factor
PP13: Placental protein 13
PPV: Positive predictive value
RIN: RNA integrity number
RIP: RNA immunoprecipitation
RISC: RNA-induced silencing complex
RNA FISH: RNA fluorescence in situ hybridization
RNAi: RNA-mediated gene silencing
ROC: Receiver operating characteristic
SD: Standard deviation
sFlt-1: Soluble fms-like tyrosine kinase-1
SiRNA: Short interfering RNA
SPE: Severe pre-eclampsia
YI: Youden index
ΔCt: Δ cycle threshold
