# Supplementary material for: Competing endogenous RNA expression profiling in pre-eclampsia identifies hsa_circ_0036877 as a potential novel blood biomarker for early pre-eclampsia
Source: Clin Epigenetics. 2018 Apr 10;10:48. doi: 10.1186/s13148-018-0482-3 (PMC5891938; doi:10.1186/s13148-018-0482-3)
Supplement: Supplementary file 1 — The demographic characteristics of the pregnant women which 12 placentas were from. The demographic characteristics of the pregnant women which blood samples were from. The top 10 differentially expressed circRNAs in placenta of PE using microarray analyses. Oligonucleotide primer sequences for qRT-PCR. Study disign. The variability between controls in microarray vs variability between PE samples was shown in BOX plot. Hsa_circ_0036877 can function as a ceRNA in the PE placenta. The correlation between the expression in placenta and maternal blood from the same individuals in the PE and non-PE grounps. (PDF 967 kb) [file 13148_2018_482_MOESM1_ESM.pdf]

Competing endogenous RNA expression profiling in pre-eclampsia identifies hsa_circ_0036877 as a potential novel blood biomarker for early pre-eclampsia

Xiaopeng Hu, Junping Ao, Xinyue Li, Huijuan Zhang, Ji Wu, Weiwei Cheng

Aditional File1

Table S1 The demographic characteristics of the pregnant women which 12 placentas were from. NS: non-significant difference.

Characteristics	Preeclampsia (n=6)	normal control (n=6)	p-value
Age (years)	31.5 ± 0.84	31.85 ± 0.98	0.5996
BMI	19.94 ± 1.19	18.82 ± 0.56	0.5012
Gestational week at delivery	35.85 ± 1.37	37.05 ± 0.31	0.0782
Proteinuria level (g/24)	2.71 ± 0.15	0	<0.05
Systolic pressure (mmHg)	168.85 ± 7.24	111 ± 3.01	0.0002
Diastolic pressure (mmHg)	114.57 ± 3.04	73 ± 3.2	<0.0001
Mode of delivery	C-section	C-section	NS
Neonatal weight (g)	2515.86 ± 263.21	3438.33 ± 122.79	0.0084
Neonatal Apgar score	10	10	NS

Table S2 The demographic characteristics of the pregnant women which blood samples were from. NS: non-significant difference.

Characteristics	Preeclampsia (n=34)	normal control (n=110)	p-value
Gestational age at sample collection (weeks)	24.8 ± 0.29	25.20 ± 0.33	0.1128
Age (years)	31.95 ± 0.59	31.49 ± 0.48	0.4673
BMI	23.46 ± 0.98	21.83 ± 0.65	0.0326
Gestational week at dilivery	37.05 ± 0.39	38.93 ± 0.33	<0.05
Proteinuria level (g/24)	0.85 ± 0.19	0	<0.05
Systolic pressure (mmHg)	142.87 ± 2.32	111.38 ± 2.42	<0.0001
Diastolic pressure (mmHG)	93.76 ± 1.33	75.43 ± 1.66	<0.0001
Cesarean section (n, %)	34 (100%)	36 (32.7%)	<0.05
Neonatal weight (g)	3023.72 ± 125.44	3517.21 ± 58.1	0.0156
Neonatal Apgar score	10	10	NS

Table S3 The top 10 differentially expressed circRNAs in placenta of PE using microarray analyses

Probe Name	T tests p-value	foldchange(abs)	regulation
hsa_circ_0055724	0.000227321	128.5154389	down
hsa_circ_0049730	5.96009E-06	49.29300736	down
hsa_circ_0001235	0.00014308	47.80985682	down
hsa_circ_0063523	6.95103E-06	46.52517171	down
hsa_circ_0063517	8.95065E-05	45.91681164	down
hsa_circ_0036474	0.00965258	20.59466442	up
hsa_circ_0065978	0.000386662	18.43054206	up
hsa_circ_0048228	0.000873199	12.61106156	up
hsa_circ_0024693	4.45253E-05	12.46826075	up
hsa_circ_0008726	0.000927737	12.27566982	up

Table S4 Oligonucleotide primer sequences for qRT-PCR.

	Forward	Reverse
Chd5	CTCAAGGCTGACGTGTTCAA	AGTGCCTCAAAGTTCCGTGT
Furin	CCGCAGATGGGTTTAATGAC	GGTCAGCGTCCCATAGTTGT
lnc-ELAVL4-9:1	TCTCATTTGGACCAGCCTCT	CTGTGGTTGCCAGCATAGTG
lnc-RAP1GAP2-5:2	TATGGTGACCTCCTGGGAAC	GTTTGAGCTGGGAAGTCGAG
hsa_circ_0036877	TTTGTAAGATGCTGGGTTGGTG	ACTGCATCTGTCACCTCGC
hsa_circ_0036878	AAACGTGCAGACTATGCAAACC	ATACTGTTGGTGTAGCCGTCG
hsa_circ_0055724	AGCCACAGAAATAAAGGATGGAGA	TCTGAGAAGACACTGGATTGCTA
hsa_circ_0049730	CCAGTCCCTACAACACCCTCA	GTAGACACTGGAAGGAGGCAAT
hsa_circ_0036474	AGACTGTTTGAATCTTGTCTCACT	TACGATCAATCACCATGGCAAC
Gapdh	CAGGAGGCATTGCTGATGAT	GAAGGCTGGGGCTCATTT

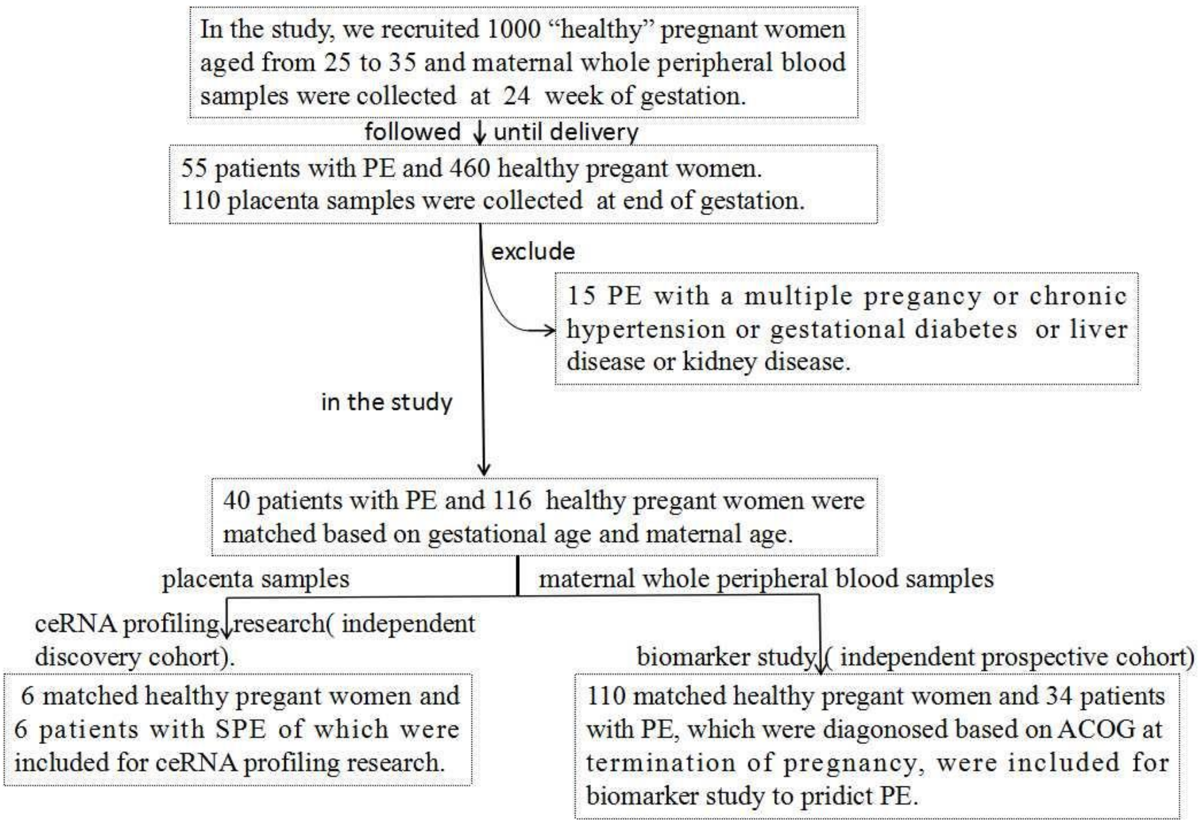

Supplement Fig.1

Supplement Fig.1: study design. This was a prospective cohort nest study and divided into two phases: ceRNA profiling research (discovery research) and biomarker study (prospective development research).

.

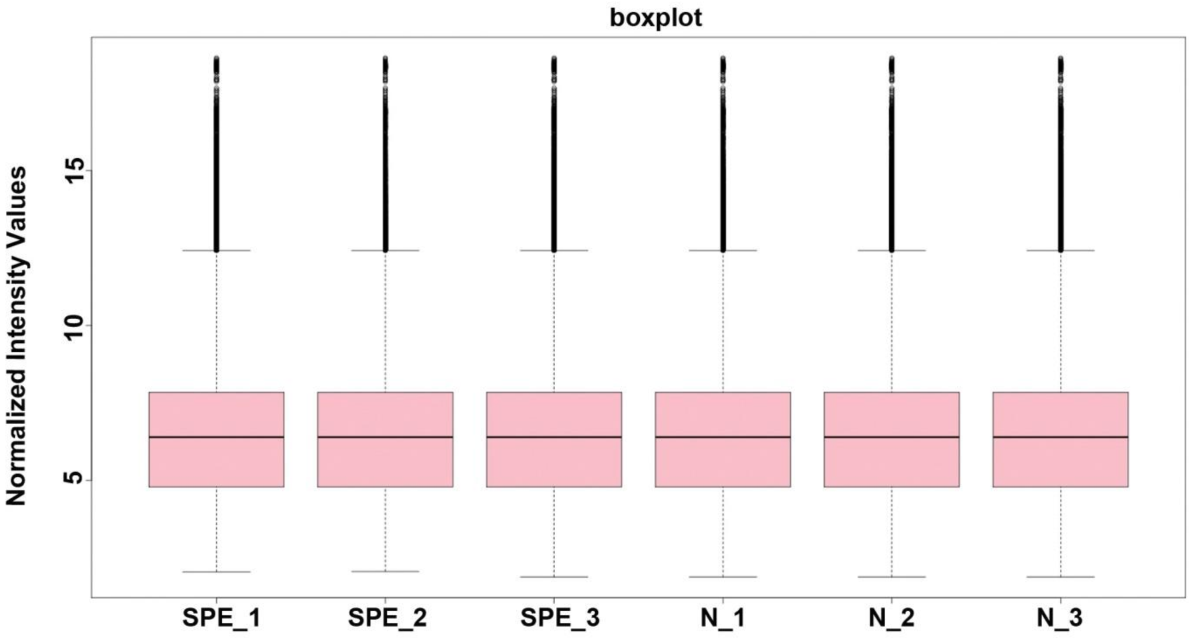

Supplement Fig.2

Supplement Fig.2: The variability between controls in microarray vs variability between PE samples was shown in BOX plot. Box plot showed the normalized intensities from SPE and N placenta tissues samples.

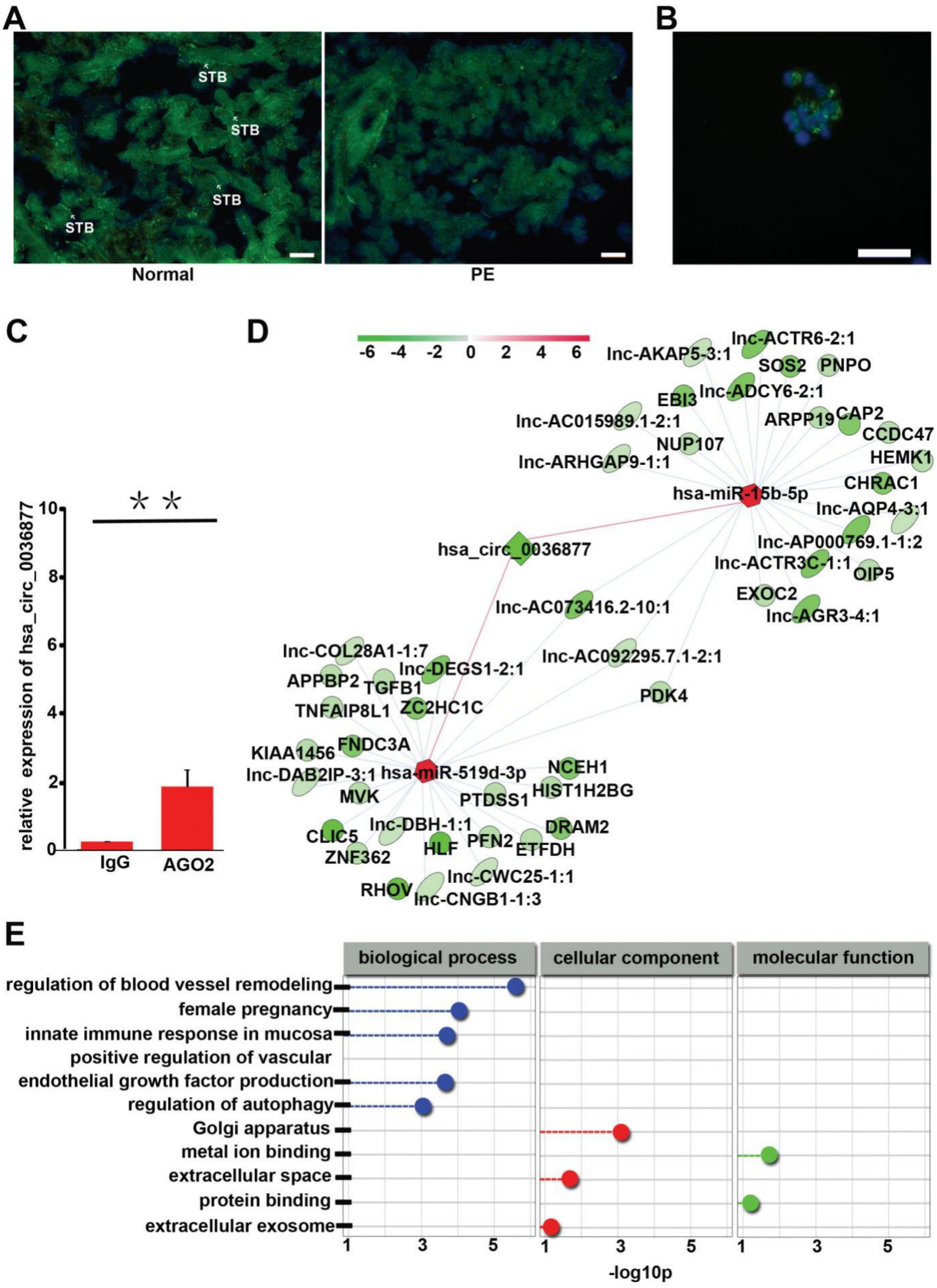

Supplement Fig.3

Supplement Fig .3: Hsa_circ_0036877 can function as a ceRNA in the PE placenta. The expression of hsa_circ_0036877 in placenta of patients with PE **(A)** and htra-8 cells **(B)** was investigated by RNA FISH. The green is positive expression and blue is DAPI**. (C)** AGO2 or rabbit IgG RNA immunoprecipitation (RIP) assays were performed in htra-8 cells. Then using qRT-PCR, the amount of hsa_circ_0036877 which AGO2 or rabbit IgG combined with was determined. Data are the means ± s.e.m. of three experiments; ***P*<0.001. **(D)** A portion of the theoretical ceRNA network of hsa_circ_0036877 in PE according to microarray analysis and report data was predicted. The color bar, which increases from green to red comparing SPE samples with matched normal controls, indicates mRNA, lncRNA, and circRNA expression levels.**(E)** GO biological processes of the above module were involved in the pathogenesis of PE. Blue nodes represent biological processes, red nodes represent cellular components, and green nodes represent molecular functions. P-values were calculated by the hypergeometric test and corrected using the Benjamini-Hochberg adjustment. P-values are expressed as negative logarithms (base 10).

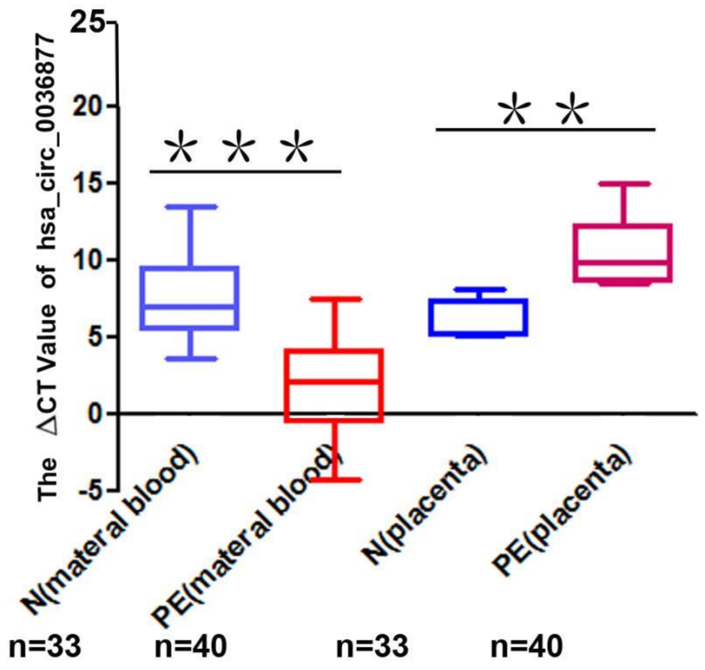

Supplement Fig.4

Supplement Fig.4 The correlation between the expression in placenta and maternal blood from the same individuals in the PE and non-PE groups.$^{**}P<0.001$,$^{***}P<0.0001$.
